# 

**Published:** 1933

**Authors:** 


					Vol. L. No. 189.
s\-: '.x?va
The Bristol
edico-Chirurgi
PUBLISHED UNDER THE AUSPICES OP
THE BRISTOL MEDICO-CHIRURGICAL SOCIETY.
Editor: 3. A. NIXON, C.M.G., M.D., F.R.C.P.
WITH WHOM ARE ASSOCIATED
E. W. HEY GROVES. M.S., F.R.C.S.. D.Sc.
A. E. FLES. O.B.E. M.B. B.S., F.R.C.S.
A. RENDLE SHORT, B.So., M.D., B.S., F.R.C.S.
E. WATSON-WILLIAMS, M.C.. Ch.M., F.R.C.S., Aanntant Rditm.
Editorial Secretary: A. L. FLEMMING. M.B.. Oh B.
??-  - - 1 ? "???,  ?. ..
SPECIAL JUBILEE NUMBER
.
VOLUME FIFTY
BRISTOL MEDICAL SCHOOL CENTENARY
!: .?:?
5
BRISTOL: J. W. ARROWSMITH LTD.. 11 QUAY STREET.
Lowdoh : J. W. Abbowsmith (London) Ltd., 8 End&leigh Gaud ens, W.0.1.
Price Three Shillings net.
BSIPMSia "* : - ?P| ' * gHgnl

				

